# Citrus Consumption and the Risk of Non-Melanoma Skin Cancer in the Women’s Health Initiative

**DOI:** 10.3390/cancers13092173

**Published:** 2021-04-30

**Authors:** Junichi R. Sakaki, Melissa M. Melough, Mary B. Roberts, Charles B. Eaton, Aladdin H. Shadyab, Abrar A. Qureshi, Ock K. Chun, Eunyoung Cho

**Affiliations:** 1 Department of Nutritional Sciences, University of Connecticut, Storrs, CT 06269, USA; junichi.sakaki@uconn.edu (J.R.S.); melissa.melough@uconn.edu (M.M.M.); 2 Brown Center for Primary Care and Prevention, Care New England Medical Group/Primary Care & Specialty Services, Pawtucket, RI 20860, USA; mary_roberts@brown.edu; 3 Department of Family Medicine, Warren Alpert Medical School of Brown University, Providence, RI 02903, USA; charles_eaton@brown.edu; 4 Herbert Wertheim School of Public Health and Human Longevity Science, University of California, San Diego, CA 92093, USA; ahshadya@health.ucsd.edu; 5 Department of Dermatology, Warren Alpert Medical School of Brown University, Providence, RI 02903, USA; abrar_qureshi@brown.edu

**Keywords:** citrus, furocoumarin, non-melanoma skin cancer, cohort, women’s health initiative

## Abstract

**Simple Summary:**

Citrus products are rich in furocoumarins, which can increase the risk of incident non-melanoma skin cancer (NMSC) when combined with ultraviolet radiation. However, few observational studies have evaluated the link between citrus intake and NMSC incidence. The aim of this study was to determine whether citrus intake was related to the NMSC incidence in participants of the Women’s Health Initiative Observational Study. The results of this study indicated that high citrus juice consumption was associated with a higher risk of incident NMSC compared to low consumption. These findings add further evidence of the potentially carcinogenic nature of certain citrus products and highlight the need to continue investigations in identifying risk factors and mechanisms.

**Abstract:**

Evidence from animal studies suggests that furocoumarins, compounds present in citrus products, can increase the risk of non-melanoma skin cancer (NMSC) when combined with ultraviolet radiation. The objective of this study was to determine the relationship between citrus intake and NMSC risk among postmenopausal women from the Women’s Health Initiative (WHI) Observational Study, who were aged 50–79 years at enrollment (1993–1998). The consumption of citrus fruit, citrus juice, and non-citrus fruit and juice were measured at the baseline of the study using a food frequency questionnaire (FFQ). NMSC cases (basal or squamous cell carcinomas) were self-reported during annual follow-up surveys. The outcome data used for this analysis were collected through March 2020. The relative risk (RR) for incident NMSC by citrus consumption was calculated. Among 49,007 non-Hispanic white participants, there were 8642 cases of incident NMSC. Using less than one serving of citrus juice per week as reference, the RRs and 95% confidence intervals (CI) for incident NMSC by citrus juice intake were 1.03 (0.95, 1.10) for one serving/week, 1.06 (1.00, 1.12) for two to four servings/week, 0.98 (0.90, 1.07) for five to six servings/week, and 1.08 (1.02, 1.13) for one or more serving/day (*p*-trend = 0.007). Subgroup analyses did not reveal meaningful associations by sun exposure variables. In conclusion, there were indications of a slightly higher risk of incident NMSC among citrus juice consumers; however, further longitudinal and mechanistic studies are needed to confirm the key risk factors.

## 1. Introduction

Non-melanoma skin cancer (NMSC), consisting primarily of basal cell carcinoma (BCC) and squamous cell carcinoma (SCC), is the most common cancer with increasing incidence rates [1,2] and predominately affects those with fair-skin [3]. While the potential for mortality is lower than that of melanoma (the mortality rates are 0.05% and 0.7% for BCC and SCC, respectively, totaling up to 2000 deaths in the US annually [4]), NMSC results in significant morbidity in the form of lesions [1], loss of function, disfigurement, and significant health care expenditures [5]. The history of NMSC increases the risk of other cancers, such as prostate, lung, breast, and colon [6,7], highlighting the importance of preventing NMSC.

There are several known risk factors for NMSC, including having a skin type that easily burns and/or tans poorly, fair complexion, increasing age, being male, and lifetime exposure to ultraviolet (UV) light [8]. UV light exposure has long been recognized as a major environmental factor for skin cancer pathology [9]. In the presence of UV radiation, naturally occurring photoactive compounds, called furocoumarins, have also been known to increase skin cancer risk [10,11,12,13,14]. Psoralen and ultraviolet A (PUVA) therapy, which uses orally or topically applied psoralen—a furocoumarin congener—in conjunction with UVA radiation to treat skin diseases, such as psoriasis, is well-known to increase the risk of skin cancer if used long term [13,15,16,17].

This raises a concern that dietary furocoumarins may potentially increase the risk of developing NMSC. Furocoumarins are largely found in citrus fruit, such as grapefruit, oranges, lemons, and limes [18,19,20]. Data from two prospective cohort studies, the Health Professionals Follow-Up Study/Nurses’ Health Study (HPFS/NHS) [21] and European Prospective Investigation into Cancer and Nutrition (EPIC) study [22] have provided epidemiological evidence indicating that citrus consumption is associated with an increased risk of incident NMSC. The participants enrolled in the HPFS/NHS were physicians and nurses, and the EPIC study participants were recruited solely in Europe, thus, highlighting the limited generalizability and representation from the US. Therefore, the objective of this study was to evaluate the association between citrus consumption and NMSC risk in a large US cohort.

## 2. Results

### 2.1. Baseline Characteristics

All measured characteristics differed between those in different categories of citrus intake. Women with higher citrus intake were older, had a higher educational level, lower BMI, and were more physically active than those consuming less citrus (Table 1). They also had greater energy and alcohol intake but consumed less coffee. With regard to sun exposure, participants who consumed more citrus tended to spend more time outdoors in the summer as a child and currently, more frequently indicated having a skin type that tans but does not burn and used a high SPF sunscreen.

### 2.2. Citrus Consumption and Risk of Incident NMSC

In total, there were 8642 cases of incident NMSC reported during an average follow-up period (mean ± standard deviation) of 14.4 ± 6.5 years. The relative risks (RR) for incident NMSC by categories of citrus intake are presented in Table 2. The highest frequency of citrus juice consumption (≥1 serving/day) was associated with a higher risk of incident NMSC in the age-adjusted model (RR (95% confidence interval) (CI): 1.12 (1.07, 1.18)) using the lowest category as reference (<1 serving/week). Adjusting for confounders, including age, BMI, education, physical activity, alcohol intake, regional solar radiation, skin reaction to sun, time spent outdoors, and sunscreen use, slightly attenuated the risk; however, the result was still significant (RR and 95% CI: 1.08 (1.02, 1.13)). Non-citrus fruit and juice intake was associated with a lower risk (highest vs. lowest category, RR and 95% CI: 0.96 (0.89, 1.03)) of NMSC in the fully-adjusted model but was not statistically significant (*p*-trend = 0.116). Neither the total citrus nor citrus fruit intake was associated with the incident NMSC risk.

To investigate the possibility of effect modification, stratified analyses were conducted with the variables that had significant interaction with citrus juice consumption and NMSC risk: regional solar radiation, skin reaction to sun, time outdoors in summer currently, and sunscreen use (Table 3). With the exception of regional solar radiation, all potential effect modifiers revealed a slightly elevated RR (1.07–1.12) for NMSC in the highest vs. lowest categories in the respective subgroups, and there was little difference in the magnitude of association. In other words, there was no difference in the RR estimates between subgroups of the effect modifiers except for regional solar radiation. For regional solar radiation, there was a higher risk of NMSC among the highest vs. lowest category of citrus juice consumption (adjusted RR (95% CI): 1.12 (1.04, 1.19)) only among those with low regional solar radiation exposure (300–350 Langleys). There was no significant association between citrus juice consumption and NMSC risk among those with high regional solar radiation exposure (375–500 Langleys).

## 3. Discussion

In this prospective cohort study, data from 49,007 postmenopausal women were used to evaluate the relation between citrus consumption and the incident NMSC risk. Citrus juice consumption was moderately associated with the NMSC risk, but non-citrus fruit and juice, citrus fruit, and total citrus consumption were not associated with NMSC risk in models adjusting for sociodemographic variables, BMI, physical activity, alcohol consumption, and sun-related variables. The stratified analyses did not reveal meaningful interactions among the subgroups of sun-related variables and NMSC.

The association between citrus intake and NMSC risk has been evaluated in few cohorts. In the EPIC study, among >250,000 participants across ten European countries, citrus juice intake was also found to be positively associated with both BCC and SCC [22]. Citrus fruit was also a risk factor for incident SCC, but not BCC, in the EPIC study, while, in our study, the total citrus fruit consumption was not associated with the risk of NMSC. In the HPFS/NHS, orange juice, but not grapefruit juice, was positively associated with the risk of incident BCC and SCC [21]. 

In both the current study and the HPFS/NHS, there was a lower risk of NMSC among participants in the highest vs. lowest categories of non-citrus fruit and juice intake. Thus, there was a pattern of opposing trends in the NMSC risk between citrus and non-citrus products, with particular concern for citrus juice. Furocoumarins are highly abundant in citrus fruit [18,19,20], including grapefruit juice and orange juice, which may contribute to the NMSC pathology [23]. Participants with a higher baseline citrus intake were then likely to have had a higher furocoumarin intake, in addition to being older and having spent more time outdoors, which may increase the NMSC risk. Observational studies investigating the risk of developing other forms of skin cancer, specifically malignant melanoma, similarly described a possible connection between citrus intake and skin cancer, although the results are equivocal [24,25,26].

In the subgroup analysis of NMSC risk factors, there was a slightly elevated risk of NMSC among subgroups for each of the four sun-related potential effect modifiers, with the exception of no significant risk among those with high exposure to regional solar radiation. These results indicate that the risk of NMSC by citrus juice consumption was not mediated by proxies of exposure to UV radiation or reaction to the sun. Others have reported an increased risk of incident melanoma or NMSC among high citrus juice consumers who spent a large amount of time outdoors in the summer [25] and had a skin type prone to burning quickly [27], which is consistent with the current hypothesis that UV radiation is a mediator between citrus consumption and NMSC risk. 

The finding that citrus juice consumption was not related to the NMSC risk among those with high regional solar radiation exposure was somewhat unexpected given our initial hypothesis that UV radiation concomitant with citrus consumption is an important mediator for NMSC risk. This may suggest that citrus juice increases the NMSC risk through non-UV-related means. However, it may simply be a spurious result arising from the lower statistical power obtained from subgroup analyses, arising from the lower number of cases within this subgroup.

The biological mechanism underlying the association between citrus juice intake and NMSC risk is likely related to the photoactivation of furocoumarins and the resulting damage to DNA. Furocoumarins are carcinogenic compounds [10,28] and are known to induce skin tumors in animals when combined with UV radiation [11,14,28]. First, furocoumarins bind to the pyrimidine base of DNA via weak bonding (hydrogen bonding and Van der Waal’s attractions) [29]. Second, irradiation with UVA light causes a cycloaddition photoreaction with the furocoumarin and the pyrimidine base, forming a monoadduct.

Subsequently, the monoadduct can absorb additional UV photons and cause the furocoumarin to bind to the thymine on the complementary DNA strand, forming a diadduct or crosslink [30]. These mono- and diadducts are capable of disrupting DNA transcription and conferring carcinogenicity [31,32,33,34]. Apart from furocoumarins’ interactions with DNA, it is possible that furocoumarins can confer carcinogenic effects from other mechanisms, such as through singlet oxygen production, lipid peroxide production, and forming adducts with protein, such as with phospholipids [15].

Interestingly, citrus fruit was not found to be associated with the NMSC risk in this study. A plausible explanation could be due to the fact that whole fruit tends to have different furocoumarin profiles compared with the juices [18,35,36]. There are furocoumarins that are uniquely abundant in the peels of citrus fruit, which are not typically consumed; however, they can migrate into the juice during the juicing process, giving juice a different furocoumarin profile [35]. Some of these furocoumarins have different carcinogenic potentials, therefore, possibly explaining the differences in cytotoxicity with respect to NMSC pathophysiology [37,38]. However, as the indexing of the carcinogenicity of the numerous furocoumarin compounds is still in its nascent stages, future investigations are needed for confirmation.

This study had several notable strengths. First, this analysis using data from the WHI Observational Study included a large sample size and long period of follow-up. Second, the cohort was well-characterized and contained detailed data on a large number of confounders, including some that are not typically measured, such as sun-related variables.

However, there were several limitations. First, the results from this study may not be generalizable to men or those who are not non-Hispanic white. Second, misclassification of skin cancer may have occurred due to the lack of adjudication for NMSC cases. Additionally, over-reporting of NMSC is possible when pre-malignant or non-cancer lesions are removed, and due to the likelihood that those with higher education (and higher citrus intake in this population) have greater access to dermatologic care. However, self-reported skin cancer data has been previously validated in other populations with high validity [39,40].

Third, there is a possibility of misclassification of exposure and covariate data. For example, while the FFQ we used was validated [41], we had dietary intake at baseline only and were not able to incorporate any changes during follow up. Similarly, the covariate information was assessed at baseline only. However, these differences would likely be non-differential and would potentially bias results towards the null. Fourth, due to the questionnaire structure, differences by NMSC type (BCC or SCC) could not be assessed.

Fifth, the FFQ grouped oranges, grapefruits, and tangerines together in one question, and therefore analyses by type of citrus fruit could not be performed. Grapefruit contains a large amount of several furocoumarin compounds, such as bergaptol, 6′7′-dihydroxybergamottin, epoxybergamottin, and bergamottin, while oranges contain a relatively small amount of bergamottin, highlighting the differences in the total furocoumarin content. The amount of total furocoumarin compounds present in grapefruit is five orders of magnitude greater than that of oranges [18]. Therefore, there may be substantial differences in the NMSC risk between citrus fruit type if this association is driven by furocoumarin content.

Finally, while our analyses were limited to non-Hispanic whites only, we lacked information on exact color of the skin and eye color to adjust for skin phototype in the analyses.

## 4. Materials and Methods

### 4.1. Study Population

The Women’s Health Initiative (WHI) is a large clinical investigation of strategies for the treatment and prevention of chronic diseases in postmenopausal women, consisting of separate clinical trial and observational study components. It enrolled participants who were aged 50–79 years at baseline from 40 clinical centers in the United States from 1993 to 1998. The division into the clinical trial and the observational study concluded in 2005, and follow-up from the observational study is ongoing. Complete details on the study design are available elsewhere [42,43,44].

For the current study, we only included participants from the Observational Study (*n* = 93,676) because those in the clinical trial lacked information on sun exposure. The data from participants available through March 2020 were used. Exclusion criteria consisted of those with a history of any cancer including NMSC (*n* = 32,311), missing food frequency questionnaire (FFQ) data (*n* = 4897), or missing sun exposure data (*n* = 10,847). Additionally, due to the relatively lower incidence of NMSC in non-white people [45], those who were not non-Hispanic white were excluded from this study (*n* = 28,267). The final analytic cohort consisted of 49,007 participants from the WHI Observational Study.

### 4.2. Citrus Consumption and Other Dietary Variables

At baseline, the participants were asked to report the frequency at which they consumed a wide variety of food and beverages over the past 3 months via an FFQ. Response options ranged from “never” to “2+ servings per day.” Total citrus consumption was considered as the sum of citrus fruit and citrus fruit juice intakes, each determined by a single FFQ item. Citrus fruit was determined by the consumption of “oranges, grapefruits, and tangerines” (one orange or half a grapefruit) while citrus fruit juice was determined by the consumption of “orange juice and grapefruit juice” (6-ounce glass).

The total non-citrus fruit and juice consumption was calculated as the sum of 12 items that contained at least partial amounts of fruit/juice: apples/pears; bananas; peaches/nectarines/plums; cantaloupes/oranges; melons/muskmelons/mangoes/papayas; watermelons/red melons; other melons; apricots; other dried fruits; strawberries/kiwis; other fruit, such as fruit cocktails/berries/grapes/applesauce/pineapples; Tang^®^/Kool-Aid^®^/Hi-C^®^/other fruit drinks; and other fruit juices, such as apple or grape. The intakes of the total energy, alcohol, and coffee were also derived from the FFQ. Nutrient estimates from the FFQ were similar to those measured from 4-day dietary records and 24-h dietary recalls in a subset of this population [41].

### 4.3. NMSC Case Ascertainment

The primary outcome of interest was the occurrence of incident NMSC during the follow-up period. The data on NMSC incidence was self-reported by participants and not adjudicated by physicians.

### 4.4. Covariates

Participants were asked to report behavioral and lifestyle information, including physical activity, education, smoking, and postmenopausal hormone use via baseline questionnaires. Data on other potential risk factors for skin cancer, including regional solar radiation (in Langleys of the participant’s clinical center), the average daily time spent outdoors in the summer as a child, the average daily time spent outdoors in the summer currently, the SPF of sunscreen used, and the skin’s reaction to the sun were collected. Weight and height were measured by trained clinical staff and were used to calculate the body mass index (BMI).

### 4.5. Statistical Analysis

Chi-square tests of association and one-way ANOVA were carried out to evaluate differences in the categorical and continuous variables, respectively, between participants in differing categories of total citrus intake.

Since incidence data on NMSC were collected annually (without the exact date of diagnosis), we were unable to conduct a Cox proportional hazards analysis. Instead, the relative risks (95% CI) were calculated for each category of citrus consumption using the lowest consumers as the referent category. The primary analysis examined the total citrus, citrus fruit, citrus juice, and non-citrus fruit, and juice intakes as the exposures of interest. Stratified analyses were conducted with the variables (dichotomized) that had significant interaction with citrus juice consumption and NMSC risk.

The initial model adjusted for age while model 1 (minimally-adjusted) additionally adjusted for BMI, education, physical activity, alcohol intake, regional solar radiation, skin reaction to sun, average daily time spent outdoors currently, and sunscreen SPF use. Model 2 (fully-adjusted) additionally included other dietary and behavioral characteristics, including total calorie intake, coffee consumption, smoking, hormone therapy status, and average daily time spent outdoors in the summer as a child. Statistical analyses were conducted using SAS software version 9.4 (SAS Institute, Cary, NC, USA). All statistical tests were two-tailed, and *p*-values < 0.05 were determined to be statistically significant.

## 5. Conclusions

In conclusion, high citrus juice consumption was moderately associated with a higher risk of NMSC in a prospective cohort of postmenopausal non-Hispanic white women. However, additional studies are needed to determine whether various NMSC risk factors modify the risk of NMSC among citrus juice consumers. Further longitudinal studies and mechanistic studies using relevant animal models are needed to elucidate the mechanisms underlying the association, particularly to identify the specific furocoumarins, foods, or beverages that may increase skin cancer risks.

## Figures and Tables

**Table 1:** Baseline characteristics of participants from the Women’s Health Initiative (*n* = 49,007) according to the frequency of total citrus consumption.

	Total Citrus Serving Category					
	<2/wk	2–4/wk	5–6/wk	1–1.4/d	≥1.5/d	Total
N (%)	13,991 (28.6)	10,870 (22.2)	5002 (10.2)	11,841 (24.2)	7303 (14.9)	
Median intake/day	0.09	0.50	0.78	1.08	1.72	
Age, years (mean, SD)	61.9 (7.1)	63.0 (7.1)	63.1 (7.2)	63.9 (7.1)	63.7 (7.3)	63.0 (7.2)
Regional solar radiation Langleys (g-cal per cm^2^) (n, %)						
300–325	4150 (29.7)	3650 (33.6)	1722 (34.4)	4388 (37.1)	2599 (35.6)	16,509 (33.7)
350	2792 (20.0)	2234 (20.6)	1060 (21.2)	2683 (22.7)	1779 (24.4)	10,548 (21.5)
375–380	1539 (11.0)	1248 (11.5)	530 (10.6)	1281 (10.8)	769 (10.5)	5367 (11.0)
400–430	2632 (18.8)	1733 (15.9)	782 (15.6)	1552 (13.1)	942 (12.9)	7641 (15.6)
475–500	2878 (20.6)	2005 (18.5)	908 (18.2)	1937 (16.4)	1214 (16.6)	8942 (18.3)
Average daily time outdoors in summer as a child (n, %)						
<30 min	338 (2.4)	239 (2.2)	104 (2.1)	245 (2.1)	159 (2.2)	1085 (2.2)
30 min to 2 h	3648 (26.1)	2975 (27.4)	1252 (25.0)	3139 (26.5)	1755 (24.0)	12,769 (26.1)
2+ h	10,005 (71.5)	7656 (70.4)	3646 (72.9)	8457 (71.4)	5389 (73.8)	35,153 (71.7)
Average daily time outdoors in summer currently (n, %)						
<30 min	4646 (33.2)	3209 (29.5)	1337 (26.7)	3366 (28.4)	2044 (28.0)	14,602 (29.8)
30 min to 2 h	6802 (48.6)	5556 (51.1)	2647 (52.9)	6160 (52.0)	3583 (49.1)	24,748 (50.5)
2+ h	2543 (18.2)	2105 (19.4)	1018 (20.4)	2315 (19.6)	1676 (23.0)	9657 (19.7)
Sunscreen SPF (n, %)						
None	7443 (53.2)	5146 (47.3)	2338 (46.7)	5532 (46.7)	3349 (45.9)	23,808 (48.6)
Something but don’t know	210 (1.5)	154 (1.4)	70 (1.4)	169 (1.4)	102 (1.4)	705 (1.4)
2–14	658 (4.7)	607 (5.6)	281 (5.6)	607 (5.1)	367 (5.0)	2520 (5.1)
15–24	3621 (25.9)	3157 (29.0)	1509 (30.2)	3626 (30.6)	2228 (30.5)	14,141 (28.9)
25+	2059 (14.7)	1806 (16.6)	804 (16.1)	1907 (16.1)	1257 (17.2)	7833 (16.0)
Skin reaction to sun (n, %)						
No change	830 (5.9)	644 (5.9)	298 (6.0)	653 (5.5)	395 (5.4)	2820 (5.8)
Tans, does not burn	4224 (30.2)	3381 (31.1)	1577 (31.5)	3652 (30.8)	2338 (32.0)	15,172 (31.0)
Burns, then tans	3600 (25.7)	2793 (25.7)	1330 (26.6)	3153 (26.6)	1933 (26.5)	12,809 (26.1)
Burns, then tans minimally	3731 (26.7)	2941 (27.1)	1303 (26.1)	3224 (27.2)	1901 (26.0)	13,100 (26.7)
Burns, does not tan	1606 (11.5)	1111 (10.2)	494 (9.9)	1159 (9.8)	736 (10.1)	5106 (10.4)
Education (n, %)						
<High school graduate	545 (3.9)	309 (2.8)	113 (2.3)	256 (2.2)	145 (2.0)	1368 (2.8)
High school graduate	2675 (19.1)	1795 (16.5)	805 (16.1)	1730 (14.6)	1001 (13.7)	8006 (16.3)
Some college	5270 (37.7)	4060 (37.4)	1681 (33.6)	4107 (34.7)	2487 (34.1)	17,605 (35.9)
College graduate	5388 (38.5)	4641 (42.7)	2375 (47.5)	5671 (47.9)	3618 (49.5)	21,693 (44.3)
BMI, kg/m^2^ (mean, SD)	27.4 (6.0)	27.1 (5.9)	27.1 (5.7)	26.7 (5.6)	26.7 (5.9)	27.0 (5.8)
Physical Activity (n, %)						
Inactive (0–1.7)	3119 (22.5)	1774 (16.5)	744 (15.0)	1788 (15.3)	961 (13.3)	8386 (17.3)
Low Activity (1.8–8.3)	3835 (27.7)	2857 (26.5)	1269 (25.6)	2892 (24.7)	1695 (23.4)	12,548 (25.9)
Mod Activity (8.4–20)	3914 (28.3)	3415 (31.7)	1559 (31.4)	3871 (33.0)	2299 (31.7)	15,058 (31.0)
High Activity (>20)	2971 (21.5)	2731 (25.3)	1387 (28.0)	3168 (27.0)	2291 (31.6)	12,548 (25.9)
Cigarette Smoking Status (n, %)						
Never	6383 (46.2)	5468 (50.9)	2523 (51.0)	6093 (52.1)	3845 (53.3)	24,312 (50.2)
Past	6346 (45.9)	4716 (43.9)	2206 (44.6)	5144 (44.0)	3070 (42.5)	21,482 (44.4)
Current	1088 (7.9)	568 (5.3)	221 (4.5)	452 (3.9)	304 (4.2)	2633 (5.4)
Alcohol, g/day (mean, SD)	5.6 (11.6)	6.1 (11.1)	6.6 (12.1)	6.6 (11.2)	6.4 (11.4)	6.2 (11.4)
Coffee, cups/day (mean, SD)	2.3 (1.8)	2.2 (1.7)	2.3 (1.7)	2.2 (1.6)	2.1 (1.7)	2.2 (1.7)
Hormone Therapy Status (n, %)						
Never	3503 (25.5)	2875 (26.9)	1317 (26.9)	3296 (28.4)	2121 (29.6)	13,112 (27.3)
Past	2653 (19.3)	1984 (18.6)	934 (19.1)	2267 (19.6)	1325 (18.5)	9163 (19.1)
Current	7600 (55.3)	5825 (54.5)	2640 (54.0)	6031 (52.0)	3711 (51.9)	25,807 (53.7)
Total energy intake, kcal (mean, SD)	1479 (567.9)	1540 (562.2)	1610 (562.3)	1626 (548.2)	1771 (604.5)	1585 (575.1)
Citrus Fruit, medium servings (mean, SD)	0.06 (0.08)	0.21 (0.18)	0.38 (0.26)	0.40 (0.37)	0.81 (0.69)	0.32 (0.42)
Citrus Juice, medium servings (mean, SD)	0.05 (0.07)	0.28 (0.19)	0.40 (0.27)	0.70 (0.38)	1.16 (0.66)	0.46 (0.51)
Non-Citrus Fruit and Juice, medium servings (mean, SD)	1.64 (1.33)	1.81 (1.23)	1.99 (1.29)	2.05 (1.36)	2.60 (1.89)	1.96 (1.44)

**Table 2:** The relative risk for incident non-melanoma skin cancer according to the frequency of citrus consumption among Women’s Health Initiative participants (*n* = 49,007).

	Serving Category					*p*-Trend
**Total Citrus**	**<2/wk**	**2–4/wk**	**5–6/wk**	**1–1.4/d**	**≥1.5/d**	
No. of cases (%)	2275 (16.3)	1903 (17.5)	873 (17.5)	2257 (19.1)	1334 (18.3)	
Age-adjusted RR (95% CI)	(ref)	1.06 (1.01, 1.13)	1.06 (0.98, 1.14)	1.15 (1.09, 1.22)	1.11 (1.04, 1.18)	<0.001
Model 1: Minimally-adjusted RR (95% CI)	(ref)	1.02 (0.96, 1.08)	1.00 (0.93, 1.07)	1.08 (1.03, 1.14)	1.03 (0.97, 1.10)	0.065
Model 2: Maximally-adjusted RR (95% CI)	(ref)	1.01 (0.96, 1.07)	1.00 (0.93, 1.07)	1.08 (1.02, 1.14)	1.02 (0.96, 1.09)	0.127
**Citrus Fruit**	**Never**	**<1/wk**	**1/wk**	**2–4/wk**	**≥5/wk**	
No. of cases (%)	1362 (16.4)	3295 (17.6)	1156 (18.2)	1257 (18.2)	1572 (18.0)	
Age-adjusted RR (95% CI)	(ref)	1.07 (1.01, 1.13)	1.09 (1.02, 1.17)	1.09 (1.02, 1.17)	1.08 (1.01, 1.15)	0.453
Model 1: Minimally-adjusted RR (95% CI)	(ref)	1.01 (0.96, 1.07)	1.00 (0.93, 1.08)	1.01 (0.94, 1.08)	0.98 (0.92, 1.05)	0.295
Model 2: Maximally-adjusted RR (95% CI)	(ref)	1.01 (0.95, 1.07)	0.99 (0.92, 1.06)	1.00 (0.93, 1.07)	0.97 (0.91, 1.04)	0.337
**Citrus Juice**	**<1/wk**	**1/wk**	**2–4/wk**	**5–6/wk**	**≥1/d**	
No. of cases (%)	3572 (16.7)	768 (17.4)	1531 (18.4)	508 (17.0)	2263 (19.0)	
Age-adjusted RR (95% CI)	(ref)	1.04 (0.97, 1.11)	1.08 (1.02, 1.14)	1.01 (0.93, 1.10)	1.12 (1.07, 1.18)	<0.001
Model 1: Minimally-adjusted RR (95% CI)	(ref)	1.02 (0.95, 1.09)	1.07 (1.01, 1.13)	0.98 (0.90, 1.07)	1.08 (1.03, 1.14)	0.002
Model 2: Maximally-adjusted RR (95% CI)	(ref)	1.03 (0.95, 1.10)	1.06 (1.00, 1.12)	0.98 (0.90, 1.07)	1.08 (1.02, 1.13)	0.007
**Non-Citrus Fruit and Juice**	**<0.75/d**	**0.75–1.2/d**	**1.3–1.9/d**	**2.0–2.9/d**	**≥3/d**	
No. of cases (%)	1321 (16.2)	1738 (17.4)	2126 (18.2)	1926 (18.3)	1531 (17.9)	
Age-adjusted RR (95% CI)	(ref)	1.06 (1.00, 1.14)	1.11 (1.05, 1.19)	1.11 (1.04, 1.19)	1.09 (1.02, 1.16)	0.029
Model 1: Minimally-adjusted RR (95% CI)	(ref)	1.02 (0.95, 1.09)	1.04 (0.97, 1.10)	1.02 (0.96, 1.09)	0.99 (0.92, 1.06)	0.440
Model 2: Maximally-adjusted RR (95% CI)	(ref)	1.01 (0.95, 1.08)	1.03 (0.96, 1.09)	1.00 (0.93, 1.07)	0.96 (0.89, 1.03)	0.116

Model 1: adjusted for age (continuous), BMI (<25.0, 25.0–29.9, 30.0–34.9, ≥35.0 kg/m^2^), education (less than high school graduate, high school graduate, some college, and college graduate), physical activity (inactive, low activity, moderate activity, and high activity), alcohol consumption (continuous), regional solar radiation (300–325, 350, 375–380, 400–430, and 475–500 Langleys), skin reaction to sun (no burns, with or without tanning; burns, and with or without tanning), average daily time outdoors currently (<30 min, 30 min–2 h, and 2+ h), and sunscreen SPF use (none, something but does not know, SPF 2–14, SPF 15–24, and SPF 25+). Model 2: Model 1 plus adjustment for the total calorie intake (continuous), coffee consumption (continuous), smoking (never, past, and current), hormone therapy status (never, past, and current), and average daily time outdoors in summer as a child (<30 min, 30 min–2 h, and 2+ h).

**Table 3:** The relative risk for incident non-melanoma skin cancer according to frequency of citrus juice consumption among Women’s Health Initiative participants (*n* = 49,007) by subgroups of potential effect modifiers.

	Citrus Juice Serving Category					*p*-Trend
	<2/wk	2–4/wk	5–6/wk	1–1.4/d	≥1.5/d	
Regional solar radiation						
300–350						
Number of cases (%)	1640 (15.1)	390 (15.9)	824 (17.1)	267 (16.2)	1321 (18.1)	
Adjusted RR (95% CI)	(ref)	1.04 (0.94, 1.15)	1.09 (1.00, 1.17)	1.03 (0.92, 1.16)	1.12 (1.04, 1.19)	0.002
375–500						
Number of cases (%)	1932 (18.4)	378 (19.2)	707 (20.3)	241 (18.0)	942 (20.3)	
Adjusted RR (95% CI)	(ref)	1.00 (0.91, 1.11)	1.05 (0.97, 1.14)	0.93 (0.83, 1.06)	1.04 (0.97, 1.12)	0.353
Skin reaction to sun						
No burn, with or without tanning						
Number of cases (%)	1094 (14.0)	218 (13.7)	488 (15.3)	163 (15.1)	683 (15.9)	
Adjusted RR (95% CI)	(ref)	0.98 (0.85, 1.12)	1.06 (0.96, 1.17)	1.06 (0.91, 1.24)	1.09 (1.00, 1.20)	0.034
Burn, with or without tanning						
Number of cases (%)	2478 (18.3)	550 (19.5)	1043 (20.4)	345 (18.1)	1580 (20.7)	
Adjusted RR (95% CI)	(ref)	1.04 (0.95, 1.13)	1.07 (1.01, 1.15)	0.95 (0.86, 1.05)	1.08 (1.02, 1.14)	0.028
Time Outdoors in Summer Currently						
<30 min/day						
Number of cases (%)	1004 (15.1)	186 (14.8)	402 (16.7)	149 (17.9)	597 (17.4)	
Adjusted RR (95% CI)	(ref)	0.95 (0.82, 1.10)	1.07 (0.96, 1.19)	1.15 (0.98, 1.34)	1.10 (1.01, 1.21)	0.015
>30 min/day						
Number of cases (%)	2568 (17.5)	582 (18.5)	1129 (19.1)	359 (16.7)	1666 (19.6)	
Adjusted RR (95% CI)	(ref)	1.04 (0.96, 1.13)	1.07 (1.00, 1.14)	0.93 (0.84, 1.03)	1.07 (1.01, 1.13)	0.040
Sunscreen Use						
No sunscreen use						
Number of cases (%)	1471 (13.6)	285 (13.9)	589 (14.8)	199 (14.0)	865 (15.6)	
Adjusted RR (95% CI)	(ref)	1.01 (0.90, 1.14)	1.06 (0.97, 1.16)	0.99 (0.86, 1.14)	1.10 (1.02, 1.19)	0.020
Sunscreen Use (any or unknown SPF)						
Number of cases (%)	2101 (19.9)	483 (20.4)	942 (21.7)	309 (19.8)	1398 (21.9)	
Adjusted RR (95% CI)	(ref)	1.03 (0.94, 1.12)	1.07 (1.00, 1.15)	0.98 (0.88, 1.09)	1.07 (1.01, 1.14)	0.042

Adjusted for age (continuous), BMI (<25.0, 25.0–29.9, 30.0–34.9, ≥35.0 kg/m^2^), education (less than high school graduate, high school graduate, some college, and college graduate), physical activity (inactive, low activity, moderate activity, and high activity), alcohol consumption (continuous), regional solar radiation (300–325, 350, 375–380, 400–430, and 475–500 Langleys), skin reaction to sun (no burns, with or without tanning; burns, and with or without tanning), average daily time outdoors currently (<30 min, 30 min–2 h, and 2+ h), and sunscreen SPF use (none, something but does not know, SPF 2–14, SPF 15–24, and SPF 25+). The stratifying variable was removed in its stratified analysis.

## Data Availability

Publicly available (upon approval) datasets were analyzed in this study. This data can be found here: https://www.whi.org/page/working-with-whi-data (accessed on 27 April 2021).
